# The Endodontic Microbial Biofilms as An Extension of Dental Plaque, a general hypothesis

**DOI:** 10.1080/20002297.2017.1325197

**Published:** 2017-06-20

**Authors:** Luis E. Chávez de Paz

**Affiliations:** ^a^ Head of Endodontics, Karolinska Institute, Stockholm, Sweden

## Abstract

Within the last years, and since the inclusion of the biofilm concept in Endodontics, our understanding of root canal infections and the periapical tissues have developed tremendously. Development of new technology and inclusion of novel clinical antimicrobial protocols nowadays rely in the fact that microbial biofilms are formed inside the root canal of teeth and thus these are main targets for elimination. However, main concepts underlying important biological aspects of biofilm formation in root canals remain unclear. In this presentation, a hypothesis is presented where the endodontic biofilm is considered as an extension of dental plaque. By means of this hypothesis, a basis for clarifying important aspects of formation, maturation and resistance of root canal biofilms are proposed.

